# Fiscal sustainability and low interest rates: what an indicator can(’t) tell

**DOI:** 10.1007/s10663-021-09531-8

**Published:** 2021-12-31

**Authors:** Martin Werding

**Affiliations:** Chair in Social Policy and Public Finance, Ruhr-Universität Bochum, Universitätsstr. 150, 44801 Bochum, Germany

**Keywords:** Public budget, Public debt, Fiscal sustainability, Interest rates, H6, J11, E43

## Abstract

The indicator that is commonly used to assess the long-term fiscal sustainability of public finances in EU member states (“*S*2”) is also defined if government borrowing rates are assumed to be permanently lower than the growth rate of GDP. Under these circumstances, however, it no longer provides a reliable orientation for fiscal policy. I illustrate these findings based on simulations prepared for the Fifth Sustainability Report published by the German Federal Ministry of Finance. In addition, I discuss the interpretation of the indicator in a low-interest environment and the assumption that relevant interest rates may continue to be low if there are substantial challenges for fiscal sustainability, *e.g.*, through demographic ageing.

**Supplementary Information:**

The online version contains supplementary material available at 10.1007/s10663-021-09531-8.

## Introduction

Applying the standard indicator for long-term fiscal sustainability that the European Commission (most recently, see European Commission 2019) and some of the member states are using to monitor fiscal policies in the EU under the Stability and Growth Pact becomes complicated if interest rates are very low or, more specifically, if the government borrowing rate is assumed to be below the growth rate of GDP not just temporarily, but permanently. In this paper, I elaborate on this observation and try to fill this gap.

The paper starts by introducing the relevant indicator (“*S*2”) and explaining why it cannot be derived in the usual fashion if the current low-interest situation lasts too long (Sect. 2). Section 3 illustrates the problem using simulations prepared for the Fifth Sustainability Report published by the German Federal Ministry of Finance (BMF 2020) and discusses whether this non-result is really harmful. In Section﻿ 4, I present an alternative approach to deriving the *S*2-indicator in a continued low-interest environment and demonstrate how it can be applied to the German example. Section 5 concludes, discussing the implications of the findings for analyses of fiscal sustainability and for long-term fiscal planning.

## The sustainability indicator *S*2

In regular reports monitoring fiscal sustainability in all member states at an EU-level, several indicators have been used to address short-term fiscal risks and fiscal sustainability in a medium-term perspective. At the same time, long-term fiscal sustainability has been consistently measured using an indicator called “*S*2” since the first EU-level report on sustainability of public finances was published (European Commission 2006). It was developed in preliminary work carried out in the years 1999 to 2003 (EU Economic Policy Committee 2001, 2003) and can be traced back to suggestions made by Blanchard (1990). The *S*2-indicator rests on simple, but highly stylized calculations, which is probably one of the reasons why this measure and the sustainability analyses using it are largely disregarded in the research literature on fiscal sustainability, where fiscal reaction functions (Bohn 1998), fiscal space (Heller 2005; Kose et al. 2017) or distributions of fiscal limits (Leeper and Walker 2011; Bi and Leeper 2013) play a core role.

The *S*2-indicator is based on the intertemporal government budget constraint which requires that—over a virtually infinite time horizon—all future public revenues must be sufficiently high to cover all future public expenditure, plus public debt that has been accumulated up to the present. In order to state this requirement formally, let $D_t$ denote public debt and $P_t$ the primary surplus (revenues minus expenditure, disregarding interest payments) in a given year $t\in \{1, 2, 3...\}$. Since nominal (and even real) amounts for both these variables are difficult to compare over time, they are usually looked at in terms of the corresponding GDP-ratios, $d_t=D_t/Y_t$ and $p_t=P_t/Y_t$. Annual GDP, $Y_t$, is assumed to grow at a (nominal) rate of $g_t$ against the preceding year. Interest payments derive from the (nominal) interest rate $r_t$ applying to government bonds. To simplify the notation, one can define a discount factor $q_t=(1+g_t)/(1+r_t)$ which converts GDP-ratios relating to period *t* into period-*t*–1 present values and assume that $q_t$ is constant from period 1 onwards, so that $q_t=q$.^1^

Given that, the intertemporal budget constraint (IBC) the government is faced with can be written as

1 $$\begin{aligned} d_0-\sum _{t=1}^{\infty } {q^t p_t} = 0, \end{aligned}$$

where period 0 is a baseline year and $t \ge 1$ are the years for which fiscal policy is to be monitored.

With real-world figures for $d_0$ and simulations regarding how $p_t$ will develop under current rules applying to revenues and expenditure, Eq. (1) often does not hold. Therefore, an improvement of future primary balances may be required to meet the IBC. Assuming that this improvement is constant in terms of its GDP-ratio and invariably applies from $t=1$ onwards leads to

2 $$\begin{aligned} d_0-\sum _{t=1}^{\infty } {q^t (p_t+\sigma )} = 0 \end{aligned}$$

as a new version of the IBC. Here, $\sigma$ is called the “sustainability gap”. It measures consolidation needs involved in the combination of $d_0$ and simulated time-series for $p_t$ in a rather stylized way, *viz.* as a single figure reflecting a constant, permanent correction that would shift the entire time path of the GDP-ratios of annual primary balances by some fraction of GDP.

For practical applications, a difficulty arises from the fact that simulations regarding the future development of $p_t$ necessarily span only a finite time horizon, until some year *T*. Lacking better information, it is therefore assumed that $p_t$ (and, if they are assumed to vary for $t<T$, also $g_t$ and $r_t$, hence $q_t$) remain constant from *T* onwards. Given that, Eq. (2) can be re-written as follows.

3 $$\begin{aligned} d_0-\sum _{t=1}^{T} {q^t p_t}-\sum _{t=T+1}^{\infty } {q^t p_T}-\sum _{t=1}^{\infty } {q^t \sigma } = 0 \end{aligned}$$

Provided that $q<1$ (because $r>g$), the sums with infinite numbers of elements included in (3) can be simplified, based on general rules for geometric series (by which $\sum _{t=1}^{\infty } {q^t}=q/(1-q)$ if $q<1$). In this case, the single terms included in each series converge towards zero as *t* approaches infinity, and the series of infinite length assume finite values. Applying this to Eq. (3), the IBC can be re-written once more, *viz.* as

4 $$\begin{aligned} d_0-\sum _{t=1}^{T} {q^t p_t} - \frac{q^{T+1}}{1-q} p_T - \frac{q}{1-q} \sigma = 0, \end{aligned}$$

which can easily be solved for the sustainability gap $\sigma$:

5 $$\begin{aligned} \sigma = \frac{1-q}{q} \left( d_0-\sum _{t=1}^{T} {q^t p_t} \right) - q^T p_T \end{aligned}$$

This is how the indicator *S*2 can be derived from the IBC under the standard assumption that $q<1$ (at least in the long run, *i.e.*, for $q_t$ with $t \ge T$).^2^ Thus far, this assumption has been considered to hold true in all scenarios that were looked at in sustainability analyses conducted at an EU-level and, for instance, also in the vast majority of scenarios covered in the German sustainability reports.

However, in a number of countries including Germany, current government borrowing rates (“$r_0$”) are below the growth rate of GDP (“$g_0$”). If this situation is assumed to continue until year *T* and even beyond, the discount factor *q* exceeds unity in the above calculations and, hence, Eq. (3) can no longer be transformed to (4) and solved for $\sigma$.

At first sight, the standard indicator for long-term fiscal sustainability, *S*2, is then no longer defined and a simple, but telling measure of consolidation needs that may arise to keep annual budget balances and the debt ratio $d_t$ on a sustainable time path is lacking. On the other hand, interest rates that are low and even fall short of GDP-growth could in themselves contribute to improving on the sustainability of public finances—again, at least at first sight. In Sects. 3 and 4, I will show that both of these assertions are premature.

## Illustrative results for Germany

Simulations prepared for the latest sustainability report of the German Federal Ministry of Finance (BMF 2020) include a number of scenarios which are explicitly meant to test the sensitivity of the results with respect to differing assumptions on future interest rates applying to German government bonds (Werding et al. 2020, pp. 123–133). Here, these scenarios are used to illustrate the implications for long-term trends in the debt ratio and for the sustainability indicator *S*2 (Sect. 3.2) and to discuss the observations (Sect. 3.3). Before doing so, a few remarks on the background of these scenarios may be needed.

### Background and underlying scenarios

Most of the work devoted to preparing simulations for the German sustainability reports is spent on projecting future time paths for public expenditure that is expected to be influenced by the on-going process of demographic ageing.^3^ Age-related expenditure in Germany^4^ amounts to almost 26% of GDP or close to 60% of total primary expenditure today. Under the current legal framework, these shares must be expected to go up in the future, since Germany is faced with an ageing process that is rather pronounced by international standards and will enter an acute phase rather soon.

For the latest German sustainability report, projections regarding future trends in age-related expenditure have been prepared using the “Social Insurance Model, version v.17” (SIM.17).^5^ Based on demographic projections—here, those provided by the Federal Statistical Office (Statistisches Bundesamt 2019) –, this model tracks the entire population, divided into age-gender-year cells, from the education system over labour-force participation to the retirement phase, with a specific focus on contributions made to financing age-related expenditure and on corresponding benefits received.

The labour force is determined based on participation rates (by age and gender) which are projected into the future using a “cohort approach” (Burniaux et al. 2003) and on unemployment rates which follow exogenous trends. The model also includes an aggregate production function that is used to simulate growth rates of labour productivity (hence, wages) and GDP. Age-related expenditure is disaggregated into age- and gender-specific per-capita figures for each of the branches of public finances covered. Relevant take-up rates and individual benefit entitlements are projected relying on plausible assumptions, intermediate results (*e.g.*, regarding changes in the population structure or wage growth) and—where they exist—current legal rules (*e.g.*, for annual pension up-ratings). The results can then be re-assembled to forming future annual aggregates.

While the EU Fiscal Sustainability Reports rest on simulations for one “baseline” scenario (combined with numerous alternative scenarios), German sustainability reports regularly provide two diverging baseline scenarios (again combined with numerous alternative scenarios). These scenarios are both based on current policies, but rest on differing assumptions affecting the age composition of the population (through higher or lower fertility, mortality and net-migration), labour-force participation and (un-)employment as well as productivity growth. Since the underlying assumptions are either basically optimistic (scenario “$T+$”) or basically pessimistic (scenario “$T-$”), but in no case extreme,^6^ the two scenarios are meant to indicate a range of possible future developments which, as of today, can be reasonably expected.

Two sets of intermediate results are particularly important here. First, building on the differing assumptions regarding demography and employment, real GDP-growth will fluctuate around 1% or around 0.5% per year over the entire simulation period in the projections relating to $T+$ and $T-$.^7^ Specifically, real growth rates for 2060 are projected to be 1.1% and 0.4%, respectively (or 3.1% and 2.4% on nominal terms, assuming a constant inflation rate of $2\%$ from 2025 onwards). Second, according to the simulations changes in the GDP-ratio of total age-related expenditure amount to +2.7 percentage points (pp) until 2040 and +3.6 pp until 2060 in the optimistic scenario $T+$. Corresponding figures for the pessimistic scenario $T-$ are +4.8 and +7.2 pp, respectively. In both cases, there is a continuous increase in this ratio throughout the simulation period, albeit at different speed and with different strength.

### Debt projections and the *S*2-indicator

Based on these intermediate results, analyses regarding the long-term sustainability of German public finances follow the same logic as in parallel work done at the EU-level. “Other” (*i.e.*, non-age-related) public expenditure is assumed to stay constant as a percentage of GDP over the entire simulation period and the same applies to public revenues. The first of these assumptions is a simplification, while the second one is a convention, or an identifying assumption, that is meant to indicate the full dimension of any sustainability problems which may be involved in current policies before entering discussions on how to bring about consolidations—either through reductions in expenditure or through higher revenues—that may be required.

Under these two assumptions, projected changes in age-related expenditure turn into changes in GDP-ratios of total primary expenditure as well as the primary balance on a one-for-one basis. In the absence of any fiscal reactions, the primary balance must therefore be expected to deteriorate by 3.6 to 7.2 pp of GDP until 2060 in the two baseline scenarios considered here. In other words, starting from a surplus of about 2% of GDP in 2019, the primary balance turns into a deficit in both these scenarios sooner or later and may become rather substantial afterwards.

**Fig. 1:**
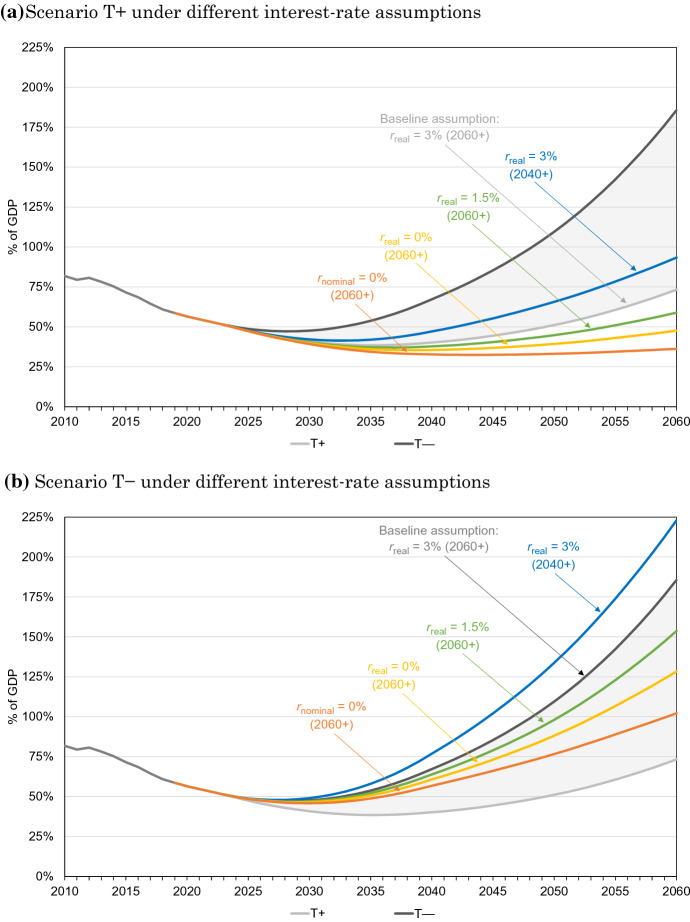
Projected debt ratios

Consequences for the total fiscal balance (including interest payments) depend on government borrowing rates and on the projected time path of government debt. Especially in cases with high interest rates, an unfavourable interaction can arise between annual budget deficits and accumulated public debt, by which both figures start to increase at accelerating speed from some point in time onwards. If the underlying increase in primary deficits is rather strong, the same can happen even if interest rates are low. This is demonstrated in Fig. 1 which exhibits simulated developments of the German debt-to-GDP ratio for the two baseline scenarios under differing assumptions regarding future government borrowing rates.

The baseline assumption regarding the interest rate for German government bonds that is uniformly applied to $T+$ and $T-$ is as follows. Starting from the current situation with a very low interest rate ($r<g$), a normalization is expected to take place over time, even though this may take quite a while. Here, “normalization” is taken to mean that the real interest rate returns to its long-term average figure from the period before the Great Recession. For simplicity, this idea is implemented through a linear transition from the most recent figure (2018: real interest rate $-0.4\%$, nominal interest rate $1.5\%$; average values for outstanding government bonds of all maturities) to the target level ($3\%$ on real terms, about $5\%$ on nominal terms) that lasts until 2060, *i.e.*, the end of the simulation period.^8^

Sensitivity tests included in Fig. 1 leave all other assumptions for scenarios $T+$ and $T-$ unchanged, but are based on alternative assumptions for the interest rate. Besides a scenario with a faster recovery (lasting only until 2040, with a constant real interest rate of $3\%$ starting from then), three scenarios with lower interest rates are considered (with linear increases of real interest rates until 2060 to 1.5% or 0%, respectively, corresponding to nominal interest rates of about 3.5% or 2%, or a linear decline to a nominal interest rate of 0%, implying a real interest rate of $-2\%$). In the latter two cases, interest rates remain below the growth rate of GDP even until 2060, so that the standard assumption of $r>g$ is violated.

Resulting trends in the debt ratio are clearly diverse (see Fig. 1). In any of the cases considered, however, debt ratios basically show a *u*-shaped pattern. The ratios go down for a while, as long as projected GDP-ratios of annual budget deficits remain below the growth rates of GDP. But as budget deficits are increasing year by year, debt ratios start to go up again at some point in time, due to the combined effects of increasing primary deficits and higher interest payments. To disentangle these effects, one may keep in mind that the time series of primary deficits are identical across all scenarios included in one of the two panels in Fig. 1, whereas interest rates differ. Therefore, the scenarios with zero nominal interest rates can be taken to mainly show the impact of increasing primary deficits (as, in these cases, interest rates start at rather low levels and decline to zero until 2060). All other scenarios then capture the consequences of higher interest rates.

The fact that the resulting debt ratios continue to go up until 2060 —mostly at accelerating speed—is a first indication of a lack in long-term sustainability. At the same time, the levels of debt are rather low until 2060 for the scenarios combining assumptions for $T+$ with low interest rates, while the debt ratio eventually increases to more than 100%, if assumptions for $T-$ are combined with interest rates permanently ranging at $r<g$.^9^

The sustainability indicator *S*2, as introduced in Sect. 2, is meant to measure consolidations (as a constant correction of the entire time series of primary balances per GDP simulated for the scenarios $T+$ and $T-$ starting from 2020 until 2060) which are required to prevent the debt ratio from increasing beyond any limit in the long run, that is, after 2060. In addition, one should keep in mind that, when calculating *S*2, primary balances, government borrowing rates and GDP-growth rates are assumed to stay constant at their year-2060 levels until infinity. Given these assumptions, the debt ratio must therefore reach a corrected level in 2060 and also stay constant afterwards to be sustainable in a long-term perspective.

For the optimistic scenario $T+$ and baseline assumptions regarding the interest rate, *S*2 turns out to be 1.5 (pp of GDP), remaining basically unchanged in the two sensitivity tests where $r>g$.^10^ For the pessimistic scenario $T-$, *S*2 becomes 4.1 under baseline interest-rate assumptions and ranges between 4.0 (if real interest rates increase to 3% until 2040) and 4.6 (if real interest rates increase to 1.5% until 2060) for the sensitivity tests with $r>g$. For scenarios in which $r<g$, the indicator *S*2 was said to be “not defined” in the study preparing the latest German sustainability report (Werding et al. 2020, pp. 130f.) for the reasons explained in Sect. 2.

### Is there a problem?

To assess whether this non-result is really harmful, one should keep in mind that the indicator *S*2 rests on calculations that are highly stylized. Specifically, the interest rate *r* (or the time path of $r_t$) is taken to be exogenously given and is not influenced by any endogenous mechanisms in a general-equilibrium model, by stochastic shocks, by the behaviour of political actors or by expectations of potential creditors—among other things, regarding the long-term sustainability of the accumulated debt level. Therefore, considering a host of possible complications that are ignored in the stylized calculations, scenarios combining strongly increasing debt ratios with permanently low interest rates may simply appear to be unrealistic.

In this sense, Andersen (2020) has recently argued that analyses of fiscal sustainability that are based on currently observed low interest rates would be misleading. His main point is that, in a situation with systematic budget deficits and, hence, increasing debt, creditors would start to ask for a credit risk premium if debt levels pass critical thresholds, so that government borrowing rates cannot remain constant. At least, uncertainty regarding such changes should be taken into account when assessing potential sustainability problems. As a consequence, sustainability analyses should always be based on interest rates that exceed the current growth rate (starting from some point in time during the simulation period and certainly in the final year), as otherwise the problems that are to be addressed are defined away. In other words, following Andersen (2020) results for *S*2 under interest rates with $r<g$ are simply not needed.

Nevertheless, the non-existence of indicator values for a situation with permanently low interest rates creates difficulties in current debates about fiscal policy. After all, a number of prominent experts have stated recently that low government borrowing rates are a reason to re-consider earlier wisdom regarding the fiscal costs of debt and the strictness of fiscal rules (see, *e.g.*, Blanchard 2019). Some experts even argue that there are fundamental reasons why the “natural” interest rate has declined and must be expected to be zero or even negative in the long run (von Weizsäcker 2017; von Weizsäcker and Krämer 2021). Many politicians tend to see this as an invitation to expand public deficits without further thinking about the long-term consequences. Against this background, it is unfortunate if a lack of results for the *S*2 indicator seemingly suggests that, in a situation with $r<g$, long-term fiscal sustainability is no longer an issue.

## Assessing *S*2 for low interest rates

In Sect. 3, it has been shown that debt ratios can reach rather high levels even if government borrowing rates continue to be very low. This is something that experts would be willing to accept who recommend to expand fiscal deficits under current interest rates, as they do not think this could be harmful. What could be surprising is that this may happen—at least in Germany—not only following a discretionary expansion of public expenditure on investment projects that might be worth consideration, but also if policy simply adheres to the current legal framework for age-related public expenditure, *i.e.*, on items which mainly serve consumptive purposes. Assessing such a situation in terms of fiscal sustainability is clearly desirable. Here, I will show that—against the impression which may have come across in Sect. 2—the *S*2 indicator can also be determined for a situation with permanently low interest rates.

### What happens if $r<g$ at $t>T$?

The nice thing about a situation with $r<g$ is that, if this lasts long enough, an economy can grow out of any level of debt, simply by reducing future deficits to zero at some point in time. According to the simulations used in Sect. 3, however, this will never happen, even not at $t=T$ or afterwards. Therefore, the debt ratio can increase to rather high levels (at least under the assumptions for scenario $T-$), even if $r<g$ (*e.g.*, if nominal or real interest rates are zero). As a consequence, creditors of fresh debt could start to ask for risk premiums leading to higher interest rates at some stage, which would accelerate further increases in the debt ratio. If $r>g$ were re-established through these risk premiums, this would openly render the situation of public finances unsustainable, as the sustainability indicator *S*2 would then confirm.

To avoid such a scenario, some orientation about fiscal consolidations that are appropriately sized and are not postponed for too long would be helpful even under the stylized assumption that $r<g$ until $t=T$ and beyond. A few candidate measures for this orientation are not fully convincing. For instance, an improvement of $p_t$ that would stabilize $d_t$ from year *T* onwards might be too less ambitious—and might also come too late. Improvements in $p_t$ from $t=1$ onwards which would limit $d_t$ to 60%^11^ until $t=T$ and keep it constant at this level afterwards are based on requirements which are rather *ad ﻿hoc*.

As a more consistent approach, one could therefore assess consolidation needs which, under the assumption of permanently low interest rates, have the same properties as a consolidation by *S*2—in cases where this indicator can be derived from the intertemporal budget constraint (see Sect. 2). To this end, we have to search for improvements in the GDP-ratio of annual primary surpluses which (*i*) become effective from $t=1$ onwards, (*ii*) are constant over time, and (*iii*) do not aim at a pre-defined level of the debt ratio at $t=T$ (or any other point in time), but will perfectly stabilize $d_t$ from year *T* onwards, as all other determinants of the debt ratio—*viz.* $p_t$ and ($r_t$, $g_t$, hence $q_t$ or) *q*—are assumed to be constant as well at $t>T$.

For an analytical solution, we start by looking at the period-0 present value of the debt ratio reached in period *T*, $d_T$, deriving from corrected primary balances, $p_t+\sigma$, from period 1 onwards. Using the same notation as in Sect. 2, it is given by

6 $$\begin{aligned} q^T d_T=d_0-\sum _{t=1}^{T} {q^t (p_t+\sigma )}=d_0-\sum _{t=1}^{T} {q^t p_t}-\sum _{t=1}^{T} {q^t \sigma }. \end{aligned}$$

For subsequent years, the period-0 present value of $d_{T+s}$, with $s\in \{1, 2, 3...\}$, becomes

7 $$\begin{aligned} q^{T+s} d_{T+s} = d_0-\sum _{t=1}^{T} {q^t p_t}-\sum _{t=T+1}^{T+s} {q^t p_T}-\sum _{t=1}^{T+s} {q^t \sigma }, \end{aligned}$$

assuming once more that $p_T$ remains unchanged for $t>T$.

In Eq. (7), the expressions $\sum _{t=T+1}^{T+s} {q^t p_T}$ and $\sum _{t=1}^{T+s} {q^t \sigma }$ can be simplified using the rules applying to geometric series, as long as $q\ne 1$.^12^ In addition, we now impose the condition that $d_t$ remains constant from period *T* onwards, so that $d_{T+s}=d_T$. This yields

8 $$\begin{aligned} q^{T+s} d_T = d_0-\sum _{t=1}^{T} {q^t p_t} -q^{T+1} \frac{1-q^s}{1-q}p_T-q \frac{1-q^{T+s}}{1-q}\sigma . \end{aligned}$$

Multiplying Eq. (8) by $(1-q)/q$ and re-arranging terms leads to

9 $$\begin{aligned} \sigma +q^{T+s} \left[ \frac{1-q}{q}d_T-p_T-\sigma \right] = \frac{1-q}{q} \left( d_0-\sum _{t=1}^{T} {q^t p_t} \right) - q^T p_T. \end{aligned}$$

Now, if the number of years after *T* included in the calculation goes to infinity, $s \rightarrow \infty$, to fully include the infinite time horizon of a comprehensive sustainability analysis, Eq. (9) behaves as follows.

1) If $q<1$ (*i.e.*, $r>g$), $q^{T+s}$ converges to zero, and $\sigma$ becomes equal to the right-hand side of (9). In other words, $\sigma$ can be determined through Eq. (5) which was already derived in Sect. 2—as expected.

2) If, instead, $q>1$ (with $r<g$), the left-hand side of (9) could diverge, because $\lim _{s \rightarrow \infty } q^{T+s} = \infty$. A necessary condition for the left-hand side of (9) to converge is that the term in square brackets is equal to zero. For $q>1$, $\sigma$ must therefore satisfy

10 $$\begin{aligned} \sigma =\frac{1-q}{q}d_T-p_T. \end{aligned}$$

If this holds true, $\sigma$ can again be assessed based on the right-hand side of Eq. (9) or based on Eq. (5). Note that Eq. (10) by no means contradicts Eq. (5) or over-determines $\sigma$. It simply states that $\sigma$ should keep the debt ratio $d_t$ constant from year *T* onwards,^13^ which is a natural property of $\sigma$ implied in (5).

3) In the case that $q=1$ (because $r=g$), some of the transformations made here are not applicable. In this case, one has to go back to Eq. (7), also using Eq. (6) to obtain

7' $$\begin{aligned} q^{T+s} d_{T+s} = q^T d_{T}-\sum _{t=T+1}^{T+s} {q^t (p_T+\sigma )}. \end{aligned}$$

Taking into account that $q=1$ and imposing the condition that $d_t$ remains constant for $t>T$, so that $d_{T+s}=d_T$, this leads to

11 $$\begin{aligned} d_T = d_{T}-s(p_T+\sigma ). \end{aligned}$$

Equation (11) implies that $\sigma =-p_T$, regardless how large *s*, which is already included as a special case in Eqs. (10) and (5). In other words, for $q=1$ the corrected primary balance needs to become zero from year *T* onwards.^14^

In any case, the main result of this exercise is that Eq. (5) is also applicable—or that the sustainability indicator *S*2 is also defined—in a situation with $q>1$ (or $r<g$).

### Illustrative application

With this new result in mind, the simulations presented in Sect. 3.2 can be taken up once again, to fill the gap in the results regarding the sustainability indicator *S*2 for scenarios with $r<g$ in 2060, *i.e.*, at the end of the simulation period. Table 1 shows the indicator values for all of the scenarios considered above.

**Table 1:** The *S*2-indicator: results for all scenarios

Interest-rate assumptions	Underlying scenarios	
	$T+$	$T-$
$r_{\text {real}}$ = 3% (2040+)	1.54	4.01
$r_{\text {real}}$ = 3% (2060+): baseline	1.49	4.10
$r_{\text {real}}$ = 1.5% (2060+)	1.48	4.55
$r_{\text {real}}$ = 0% (2060+)*	1.52	5.39
$r_{\text {nominal}}$ = 0% (2060+)*	1.82	8.63

Annotations: All figures are measured as a percentage of GDP, indicating permanent improvements in annual primary balances of the general-government budget which are required starting from 2020 to meet the intertemporal government budget constraint over an infinite time horizon.

*Scenarios with $$r<g$$.

Sources: SIM.17 (Werding et al. 2020); own calculations

Two points are remarkable about the amended set of results for *S*2 displayed in the table. First, since assumptions on interest rates are arranged in descending order—meaning that interest rates tend to become lower in each new row—it is now visible that the sustainability gap measured by *S*2 gets smaller as *r* decreases only if primary deficits simulated for year *T* are relative low (as they are under the assumptions for the optimistic scenario $T+$) and if interest rates are normal (with $r>g$). In any other case, *S*2 increases with declining interest rates.

This property of the *S*2-indicator is already well-known. It is due to the fact that closing the sustainability gap through corrections of the primary balance from year 1 onwards is effectively based on a strategy of pre-funding for future deficits. With lower interest rates, this strategy becomes more difficult. Alternatively, the same effects can be explained by the fact that, when assessing *S*2, interest rates influence the present-value weights of future deficits. With lower interest rates, high primary deficits accruing in the more remote future become more important, as they are discounted less heavily. This is nicely demonstrated in the recent contribution by (Andersen (2020), pp. 32f.).

Second, the results summarized in Table 1 also reveal that the indicator *S*2, which exhibits a relatively low sensitivity with respect to *r* as long as $r>g$,^15^ becomes highly sensitive to government borrowing rates if the latter are assumed to be very low (with $r<g$). In fact, in the cases with very low interest rates, the *S*2 indicator tends to overshoot—considering the fact that primary deficits simulated for the year 2060 (which are assumed to remain constant afterwards) are 1.5% of GDP in the scenario $T+$ and 5.1% of GDP in scenario $T-$.

In the optimistic scenario, long-term fiscal sustainability essentially requires to bring the primary balance close to zero from 2060 onwards, as long as nominal interest rates are positive. But if the interest rate is assumed to fall to (or below) 0% on nominal terms until 2060, *S*2 seems to suggest a stronger consolidation. In the pessimistic scenario, consolidations needed to render public finances sustainable in the long run even allow for a (corrected) primary deficit from 2060 onwards, as long as $r>g$. In these cases, early consolidations by *S*2 make sure that, until 2060, the state holds a sufficient amount of public wealth (not debt) to cover the remaining primary deficit by earned interest.^16^ With very low interest rates, however, this is possible only under extreme forms of accumulation of public wealth which do not appear to be sensible. At the same time, fiscal costs arising from high levels of public debt arising in the absence of any consolidation become a problem only if government borrowing rates return to normal levels. In this case, however, smaller steps to consolidation would be sufficient, as is indicated by the results for *S*2 under assumptions with $r>g$.

The main lesson to take away from this illustration is therefore the following. While it is indeed possible to determine the *S*2-indicator for long-term fiscal sustainability for scenarios with $r<g$ (as shown in Sect. 4.1), the results can be interpreted only with an eye on the underlying simulations for primary balances and debt ratios without any fiscal consolidations (see Fig. 1). If primary deficits expected towards the end of the simulation period are low, because projected increases in age-related public expenditure are relatively small, and if the debt ratio stays well below its current level over the entire time horizon, due to very low government borrowing rates, sustainability problems involved in current legal rules are not very pressing. In such a case, that is, under the optimistic assumptions for scenario $T+$, consolidation could possibly be postponed until interest rates show signs of a recovery.

If, on the other hand, the primary deficit projected for year *T* is substantial and if the debt ratio clearly exceeds the current level towards the end of the simulation period even under $r<g$ (and would continue to increase indefinitely afterwards), the situation is different. In this case, *e.g.*, under the assumptions for scenario $T-$, fiscal consolidation is definitely called for. However, the dimension of consolidations needed to render public finances sustainable would be overstated by figures for *S*2 relating to a permanent low-interest-rate environment. Instead, results for the *S*2-indicator under assumptions with $r>g$ offer a reliable orientation for the size of fiscal reactions which are required. Assessing the sustainability gap also for situations with $r<g$ is therefore useful, as it may help to avoid possible misperceptions. At the same time, Andersen (2020) is right in saying that precise results for *S*2 under $r<g$ are not of interest because, in themselves, they are not very telling.

One way of dealing with these insights could be to revise the procedures applied when assessing *S*2. In these calculations, government borrowing rates could be endogenized—*e.g.*, by directly linking them to the debt ratio –, so that they automatically adjust to normal values, if the debt ratio exceeds some critical threshold. Before moving in this direction, however, more empirical work may be needed to clarify whether such a modelling approach appears to be realistic and, if so, whether the reactions would be strong enough to make sure that $r>g$ in period *T*, at least in unfavourable scenarios such as $T-$.

## Conclusions

All observations made in Sects. 3 and 4 effectively point in the same direction. When it comes to assessing the long-term sustainability of public finances, assumptions regarding future trends of government borrowing rates are of secondary importance. The current situation with low interest rates, and even the expectation that it may last for quite some time, does not provide good news, if primary balances must be expected to deteriorate substantially over the next decades as the ageing process unfolds. Conversely, if interest rates start to increase again at some point in time in the future, the situation of public finances does not get worse than it already is. What needs to be done to make public finances sustainable is fully captured by results for *S*2 under assumptions with $r>g$. Also, in such a “normal” constellation, sensitivity of the indicator with respect to the precise level of *r* is not very strong.

Higher results for *S*2 which are obtained under assumptions with $r<g$ are a kind of warning sign. They should remind those in charge of fiscal policy that, when they exist, problems regarding the long-term sustainability of public finances are not removed through low government borrowing rates. There is still a need for correcting primary deficits that drive up the debt ratio. Otherwise, borrowing rates may normalize through risk premiums that creditors would ask for, even if fundamental reasons for “safe” interest rates to be permanently low apply. In other words, being considered a “safe haven” is not guaranteed if—for instance, in the case of Germany —a country is faced with an ageing process that is more pronounced than elsewhere and if age-related public expenditure must be expected to increase considerably under the existing legal framework.

For practical fiscal planning, implications of the results obtained here are limited, but not entirely negligible. Closing the long-term sustainability gap *S*2 has never been considered a hard fiscal rule, due to uncertainties involved in the underlying simulations and the highly stylized nature of the calculations it rests on. For instance, the “medium-term objectives” set in the year-2012 European Fiscal Compact are not influenced by the results which are regularly published in the EU Fiscal Sustainability Reports. Current discussions about the “debt brake” installed in Germany and a number of other European countries, which are fueled by expectations of long-lasting low interest rates, typically refer to needs for public investment—mainly, to limit climate change and promote the process of digitization—that a number of discussants consider (even) more important than balanced budgets and sustainable debt levels. While this view is clearly debatable,^17^ making permanent exemptions from existing fiscal limits for public consumption expenditure (on pensions, health care, unemployment, *etc.*) should continue to be ruled out, not at least due to the uncertainties regarding future interest rates.

If, however, current fiscal rules are to be replaced by less well-defined “fiscal standards” based on stochastic debt sustainability analyses (as is suggested by Blanchard et al. 2020), taking into account that future trends in age-related expenditure have systematic, not purely stochastic, components may well be required. How this can be fitted together may deserve further thinking. Stochastic population projections have long become a standard in demographic research (see, *e.g.*, Lee 1992). Fully capturing the uncertainties relating to other determinants of future age-related expenditure—such as labour-force participation, trend unemployment, productivity growth, retirement behaviour, morbidity, *etc.*, plus future changes in relevant legal rules—in a stochastic framework may turn out to be difficult, though. In any case, where the consequences for future expenditure must be expected to be strong and unfavourable, they ought to have an impact on planning and monitoring fiscal policy already in the short to medium run.

In addition, the considerations made here also have implications for the research agenda of those who are interested in budget planning and fiscal sustainability. Specifically, further research may be needed regarding the nature of trend reversals in government borrowing rates. Thus far, it has been demonstrated that reversals of this kind occur with much regularity and that, currently, a reversal indeed appears to be delayed.^18^ More and in-depth empirical work may be needed regarding the causes of interest-rate reversals as well as the determinants of risk premiums related to government bonds, capturing not only the role of deficits and debt-to-GDP ratios, but also the structure of debt (*e.g.*, by types of creditors or by currencies in which debt is denominated), the existence and ideally also the strictness of fiscal rules, and other aspects of the governance of fiscal policy and debt management.

## Supplementary Information

Below is the link to the electronic supplementary material.

Supplementary file1 (XLSX 76 kb)

## Data Availability

Not applicable.

## Footnotes

1 In the illustrative simulations considered in Sects. 3 and 4, both $g_t$ and $r_t$ may vary over the entire simulation period.

2 The formal derivation of *S*2 provided in (European Commission (2019), annex A2.4) looks much more complex but is mathematically equivalent if one accepts a few simplifications made here.

3 This work parallels the preparation of the EU Ageing Reports (most recently, see European Commission and EU Economic Policy Committee 2018) which regularly precede the EU Fiscal Sustainability Reports.

4 This includes public expenditure on old-age provision (Statutory Pension Scheme, civil servants’ pensions), health and long-term care (Statutory Health Insurance, Social Long-term Care Insurance, similar benefits for civil servants and their family members), education (pre-primary through tertiary), core measures of family policies and—due to the important role of employment for financing all other items on a pay-as-you-go basis – expenditure on unemployment and measures of active labour-market policies.

5 For a documentation of data bases used and modelling strategies applied, see Werding (2013).

6 Assumptions and numerous results are documented in Werding et al. (2020, ch. 2). An English summary covering some of these materials can be provided upon request.

7 Note that the simulations presented here were essentially prepared in the second half of 2019. Therefore, they do not include any effects of the current covid-19 pandemic on GDP and many other items considered.

8 Instead of a linear transition, a further decline with subsequent reversal could be more realistic, even in the case of a long-term normalization, since the current market rate for fresh debt is well below the average figure indicated above. Also, one might question the return to pre-2008 figures and choose a lower target level. For the considerations made here, all this is of secondary importance.

9 Again, the simulations presented here do not reflect the effects of the covid-19 pandemic on public budgets. In 2020, the German debt ratio has jumped to 69.8%, by about 10 pp. Even in the case of a quick recovery of the German economy, but in the absence of specific consolidation measures, this would shift all curves shown in Fig. 1 upward and speed up their increase towards 2060.

10 Rounded figures are 1.5 again, with some variation at the second digit of more precise results. For a full overview over these and further results see Table 1 in Sect. 4.2 below.

11 Corrections of primary balances that limit the debt ratio to a maximum of 60% for differing time horizons have been used as “*S*1”-indicators of fiscal sustainability in most of the EU-level sustainability reports.

12 For finite series, these rules are applicable also if $q>1$.

13 This can easily be seen, if Eq. (10) is re-written, replacing *q* with $(1+g)/(1+r)$. It follows that $-(p_T+\sigma )=(g-r)d_T/(1+g)$. Here, $-(p_T+\sigma )$ is the corrected primary deficit, after consolidation, accruing in any year $t>T$; $d_T/(1+g)$ is the debt ratio of the preceding year adjusted to annual GDP-growth; current interest payments on existing debt amount to $rd_T/(1+g)$ on a per-GDP basis; to keep the debt ratio constant, a total deficit ratio corresponding to $gd_T/(1+g)$ can be incurred in the new year, making sure that public debt grows exactly as fast as GDP; a correction of projected figures for $p_T$ through $\sigma$ may therefore be needed to meet this condition.

14 In this case, interest payments per GDP of $rd_T/(1+g)$ create a fiscal deficit in each year $t>T$ that makes public debt increase exactly in line with GDP-growth, *viz.* at rate *g*.

15 Under this assumptions, changes in *S*2 are negligible for the variants on scenario $T+$, and even the range of results deriving from the pessimistic scenario $T-$ appears small compared to changes in projected increases in future debt ratios that are associated with the differing interest-rate assumptions considered here (see Fig. 1 above).

16 To keep the (negative) debt ratio constant, part of the interest earnings must also be used to adjust $D_{T+x}$ in line with GDP-growth. Total annual surpluses—deriving from primary deficits plus interest earnings—must therefore equal increases in public debt (or wealth) by the growth rate of GDP, $-gD_{T+x-1}$ (see footnote 13).

17 Only, those advocating it should be reminded that the main reason why strict limits such as the debt brake were adopted was the practical impossibility of providing definitions of public investment that are sufficiently clear and distinct to avoid any circumventions of more flexible rules.

18 See, for instance, Mehrotra (2017), building on the database compiled by Jordá et al. (2020). Further work that is of interest here has been carried out by Sergeyev and Mehrotra (2019) or Lian et al. (2020), based on calibrated simulations and on regressions of the $r-g$-differential on the debt ratio and a few macroeconomic variables, respectively.
